# Steady Laminar Flow Decreases Endothelial Glycolytic Flux While Enhancing Proteoglycan Synthesis and Antioxidant Pathways

**DOI:** 10.3390/ijms25052485

**Published:** 2024-02-20

**Authors:** Sarah E. Basehore, Jonathan Garcia, Alisa Morss Clyne

**Affiliations:** 1 School of Biomedical Engineering, Science, and Health Systems, Drexel University, Philadelphia, PA 19104, USAjonathangarcia.ua@gmail.com (J.G.); 2 Fischell Department of Bioengineering, University of Maryland, College Park, MD 20742, USA

**Keywords:** endothelial cell, metabolism, glycolysis, hemodynamics, shear stress, disturbed flow, proteoglycans, oxidative stress

## Abstract

Endothelial cells in steady laminar flow assume a healthy, quiescent phenotype, while endothelial cells in oscillating disturbed flow become dysfunctional. Since endothelial dysfunction leads to atherosclerosis and cardiovascular disease, it is important to understand the mechanisms by which endothelial cells change their function in varied flow environments. Endothelial metabolism has recently been proven a powerful tool to regulate vascular function. Endothelial cells generate most of their energy from glycolysis, and steady laminar flow may reduce endothelial glycolytic flux. We hypothesized that steady laminar but not oscillating disturbed flow would reduce glycolytic flux and alter glycolytic side branch pathways. In this study, we exposed human umbilical vein endothelial cells to static culture, steady laminar flow (20 dynes/cm^2^ shear stress), or oscillating disturbed flow (4 ± 6 dynes/cm^2^ shear stress) for 24 h using a cone-and-plate device. We then measured glucose and lactate uptake and secretion, respectively, and glycolytic metabolites. Finally, we explored changes in the expression and protein levels of endothelial glycolytic enzymes. Our data show that endothelial cells in steady laminar flow had decreased glucose uptake and ^13^C labeling of glycolytic metabolites while cells in oscillating disturbed flow did not. Steady laminar flow did not significantly change glycolytic enzyme gene or protein expression, suggesting that glycolysis may be altered through enzyme activity. Flow also modulated glycolytic side branch pathways involved in proteoglycan and glycosaminoglycan synthesis, as well as oxidative stress. These flow-induced changes in endothelial glucose metabolism may impact the atheroprone endothelial phenotype in oscillating disturbed flow.

## 1. Introduction

Cardiovascular disease remains the leading cause of death in the United States and worldwide. Cardiovascular disease is a metabolic disease. Metabolic risk factors, including elevated triglycerides, obesity, high-density lipoprotein (HDL), and elevated fasting blood sugar contribute to the endothelial cell dysfunction that precedes atherosclerotic plaque development [1,2]. While widely used therapeutics target these metabolic abnormalities, there are few therapeutics that target cardiovascular disease caused by altered metabolism.

Cardiovascular disease is also a hemodynamic disease. Endothelial cells that are in direct contact with flowing blood are exposed to shear stress created by frictional forces between the blood and the vessel wall [3,4]. Blood flow in straight arterial sections is steady and laminar, with shear stress magnitudes ranging from 10 to 70 dynes/cm^2^ [5]. In contrast, blood flow near arterial bifurcations and curvatures is disturbed and re-circulatory or oscillating, with a lower net shear stress magnitude (less than 4 dynes/cm^2^) [6]. Steady laminar flow promotes a healthy, quiescent endothelial phenotype. However, in areas of oscillating disturbed flow, endothelial cells become dysfunctional [7,8]. Disturbed flow areas are linked to atherosclerosis [9].

While endothelial cells are exposed to both metabolic factors and hemodynamic forces, little is known about how hemodynamics regulates endothelial cell metabolism. Endothelial cells are highly glycolytic, even in the presence of oxygen [1,10]. Glucose enters endothelial cells through a glucose transporter (GLUT1), after which it is converted to glucose 6-phosphate (G6P) and then fructose 6-phosphate (F6P). F6P conversion to fructose-1,6-bisphosphate by 6-phosphofructo-1-kinase (PFK-1) is a key glycolysis checkpoint [11] that is regulated by phosphofructokinase-2/fructose-2,6-bisphosphatase-3 (PFKFB) [12]. Glucose ultimately breaks down into pyruvate, which is then either converted to lactate to exit the cell or shuttled into the mitochondria for oxidative phosphorylation in the tricarboxylic acid (TCA) cycle. Glycolysis intermediates are also used in metabolic pathways that branch off glycolysis, including the pentose phosphate pathway (PPP), hexosamine biosynthetic pathway (HBP), glycogen synthesis, and the uronic acid pathway. These pathways are key regulators of redox balance, nucleotide synthesis, posttranslational protein glycosylation, macromolecule biosynthesis, and intracellular metabolism [13].

Both in vivo and in vitro studies suggest that glycolysis may be important in atherosclerosis. Both glucose uptake and glycolysis were increased in atherosclerotic-like lesions in rabbits and apolipoprotein E-deficient (ApoE−/−) mice [14,15]. In endothelial cells specifically, elevated lipoprotein levels increased glycolysis in human arterial endothelial cells, and this effect was abrogated by PFKFB3 inhibition [16]. Thus, there is potential to target glucose metabolism for preventing or treating atherosclerosis and cardiovascular disease.

Many studies on endothelial cell metabolism focused on PFKFB3 [12,17,18], which controls angiogenic sprouting through its role in glycolysis [12,19,20,21]. More recently, PFKFB3 expression was shown to decrease in endothelial cells exposed to 72 h of laminar shear stress (20 dynes/cm^2^) via the flow-sensitive transcription factor KLF2 [22]. While KLF2 overexpression decreased PFKFB3 and glycolytic flux, concurrent KLF2 and PFKFB3 overexpression only slightly increased endothelial glycolytic flux. These data suggest that other glycolytic mechanisms may be regulated by laminar shear stress. Additionally, glucose metabolism in endothelial cells exposed to oscillating disturbed flow has not been extensively studied, despite the known effects of disturbed flow on vascular health [5,6].

We hypothesized that steady laminar flow, but not oscillating disturbed flow, would decrease glycolytic activity, contributing to a healthy, quiescent endothelial cell phenotype. In support of this hypothesis, we used a cone-and-plate device to adapt endothelial cells to steady laminar and oscillating disturbed flow for 24 h. We then measured endothelial glycolytic flux via a YSI bioanalyzer and mass spectrometry. We further measured glycolytic enzyme expression via RT-PCR as well as protein levels via Western blot. Overall, our data show that glucose metabolism is differentially regulated by steady laminar and oscillating disturbed flow. These metabolic changes could, in turn, impact endothelial dysfunction in disturbed flow.

## 2. Results

**Figure 1:**
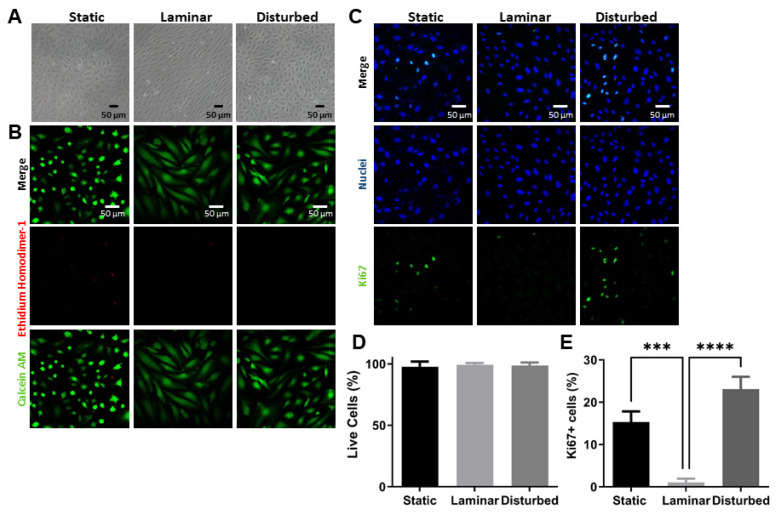
HUVECs exposed to steady laminar flow in the cone-and-plate device aligned in the flow direction and had decreased proliferation as compared to HUVECs in static culture or oscillating disturbed flow. Representative images of HUVECs after 24 h of static culture, steady laminar flow, or oscillating disturbed flow assessed for (**A**) cell alignment by phase contrast microscopy; (**B**) cell viability using calcein-AM (green, live cells) and ethidium homodimer-1 (red, dead cells) by confocal microscopy; and (**C**) proliferation using Ki67 (green, proliferation marker) and Hoechst 33,342 (blue, nuclei) by confocal microscopy. Scale bar = 50 µm. (**D**) Percentage of live cells and (**E**) percentage of Ki67+ cells were then quantified using ImageJ via the cell counter analyzer plugin. *n* = 3 samples per condition. *** *p* < 0.0001, **** *p* < 0.00001.

We first determined that the cone-and-plate device recapitulated the established endothelial cell responses to steady laminar and oscillating disturbed flow (Figure 1). Indeed, human umbilical vein endothelial cells (HUVECs) cultured in 24 h of steady laminar flow aligned and elongated in the flow direction, while those in static culture or oscillating disturbed flow remained in a typical cobblestone morphology (Figure 1A). We then quantified the number of viable and proliferating cells using confocal microscopy and ImageJ. Nearly 100% of HUVECs were viable in static culture, steady laminar flow, and oscillating disturbed flow (Figure 1B,D). Out of an average of 50 cells per image, less than 5 cells in any image were identified as dead. Between 12 and 26% of HUVECs in static culture and in oscillating disturbed flow were proliferative (around 8 out of 50 cells per image in static culture, and around 20 out of 85 cells per image in oscillating disturbed flow), while less than 2% of HUVECs in steady laminar flow were proliferative (less than 1 out of 65 cells per image in steady laminar flow), as measured via Ki67 labeling (Figure 1C,E). These results are consistent with previous reports of endothelial phenotype in steady laminar and oscillating disturbed flow and thus indicate that the cone-and-plate device effectively models these two different types of flow [23].

**Figure 2:**
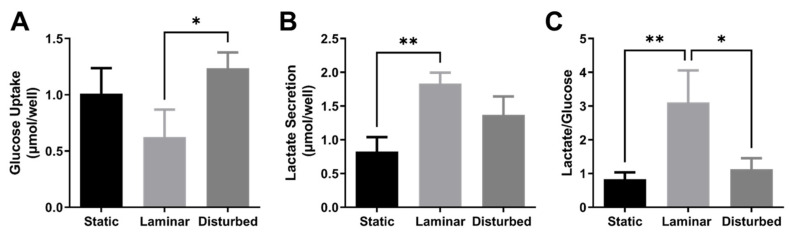
Glucose consumption was lower in HUVECs exposed to steady laminar flow compared to oscillating disturbed flow. (**A**) Glucose uptake and (**B**) lactate secretion in HUVECs exposed to 24 h static culture, steady laminar flow, or oscillating disturbed flow, measured via a YSI bioanalyzer. (**C**) Lactate/glucose ratio in HUVECs exposed to static culture, steady laminar flow, or oscillating disturbed flow. *n* = 3 samples per condition. * *p* < 0.01, ** *p* < 0.001.

We then used a YSI bioanalyzer to obtain an overview of glycolytic activity in endothelial cells exposed to flow. Glucose uptake was nearly two times higher in HUVECs exposed to oscillating disturbed flow (*p* < 0.01) as compared to steady laminar flow (Figure 2A). In contrast, HUVECs exposed to steady laminar flow had nearly two times higher lactate secretion (*p* < 0.001) compared to cells in static culture, despite less glucose uptake (Figure 2B). HUVECs exposed to steady laminar flow released around three times more lactate per glucose molecule consumed (*p* < 0.001, Figure 2C) than HUVECs in either static culture and oscillating disturbed flow.

**Figure 3:**
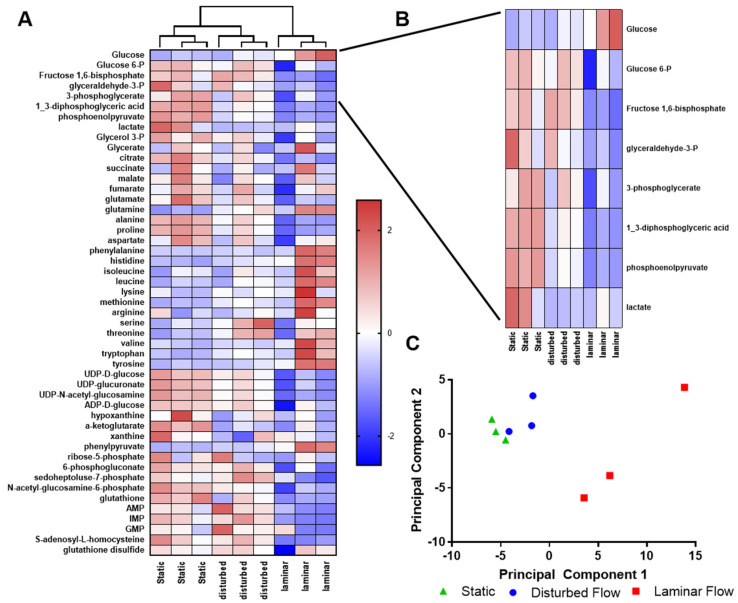
Endothelial cells exposed to steady laminar flow were metabolically distinct from cells in static culture or oscillating disturbed flow. (**A**) Heat map of metabolite total pools for HUVECs exposed to static culture, steady laminar flow, or oscillating disturbed flow for 24 h, as measured by LC-MS. Blue indicates smaller pool size whereas red indicates larger pool size. (**B**) Heat map of only glycolytic metabolites in HUVECs exposed to static culture, steady laminar flow, or oscillating disturbed flow. (**C**) PCA of glycolytic metabolites in HUVECs exposed to static culture (green), steady laminar flow (red), or oscillating disturbed flow (blue). *n* = 3 samples per condition.

To obtain more detail on endothelial glucose metabolism, we treated cells exposed to different flow regimes for 24 h with U-^13^C_6_-glucose for 5 min and then analyzed glucose-derived metabolites by liquid chromatography–mass spectrometry (LC-MS). Most of the measured total metabolite pools were lower (blue) in HUVECs exposed to steady laminar flow as compared to those in static culture or oscillating disturbed flow (Figure 3A). The exception was that many amino acids had larger total pools for HUVECs in steady laminar flow (red). When we examined only the glycolytic metabolites (Figure 3B), all metabolite total pools (except glucose) were lowest in HUVECs exposed to steady laminar flow. These differences were confirmed using principal component analysis (PCA; Figure 3C), in which static culture (green) and oscillating disturbed flow (blue) samples clustered together but steady laminar flow (red) samples were separate, especially across principal component 1.

**Figure 4:**
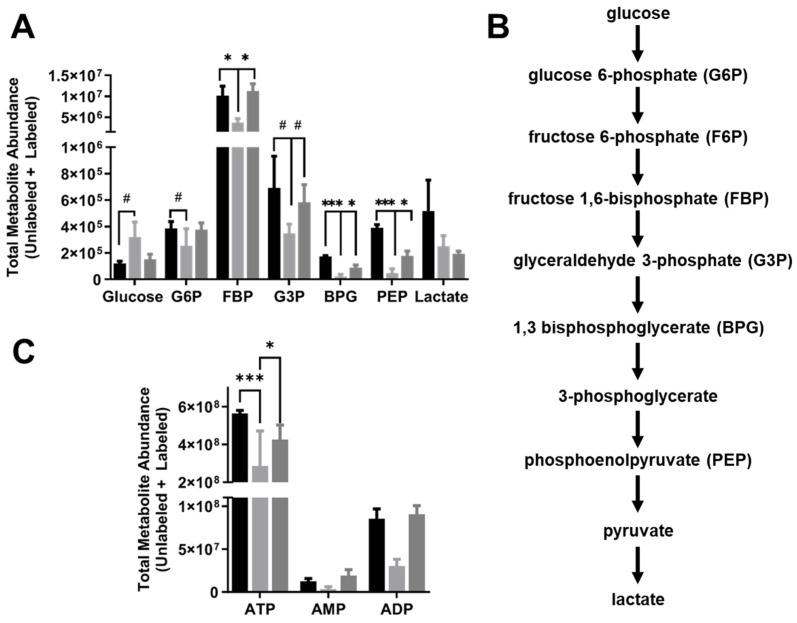
HUVECs adapted to steady laminar flow had lower total glycolytic metabolite abundances. (**A**) Total glycolytic metabolite abundances (labeled + unlabeled) for HUVECs exposed to static culture, steady laminar flow, or oscillating disturbed flow for 24 h and then labeled with U-^13^C_6_-glucose for 5 min. (**B**) Glycolytic pathway diagram showing glucose metabolism into lactate. (**C**) Total ATP, AMP, and ADP abundances (labeled + unlabeled). *n* = 3 samples per condition. # *p* < 0.05, * *p* < 0.01, *** *p* < 0.0001.

**Figure 5:**
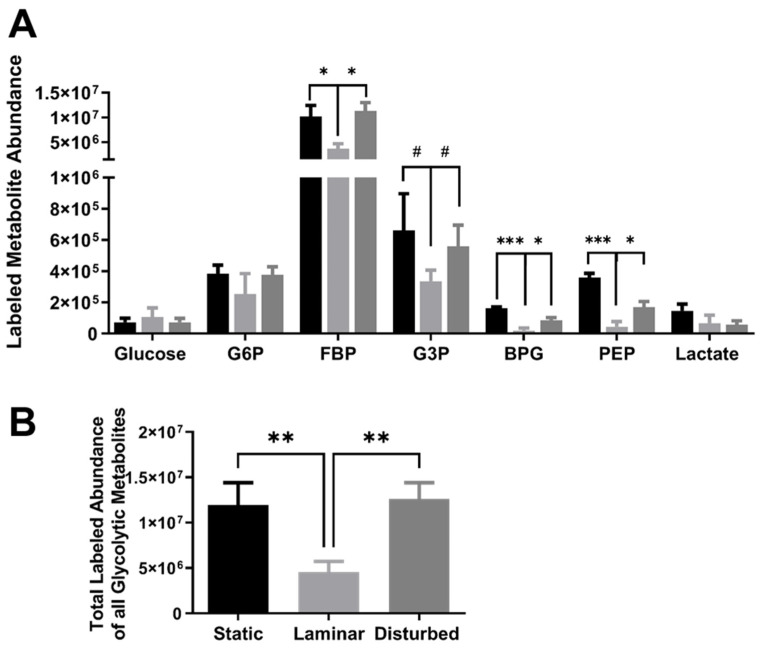
HUVECs adapted to steady laminar flow had lower labeled glycolytic metabolite abundances. (**A**) Labeled metabolite abundances and (**B**) total labeled metabolite abundance, calculated as the summation of all labeled glycolytic metabolite abundances. HUVECs were exposed to static culture, steady laminar flow, or oscillating disturbed flow for 24 h and then labeled with U-^13^C_6_-glucose for 5 min. Measured metabolites include glucose, glucose-6-phosphate (G6P), fructose 6-bisphosphate (FBP), glyceraldehyde-3-phosphate (G3P), 1,3-bisphosphoglycerate (BPG), phosphoenolpyruvate (PEP), and lactate. *n* = 3 samples per condition. # *p* < 0.05, * *p* < 0.01, ** *p* < 0.001, *** *p* < 0.0001.

**Figure 6:**
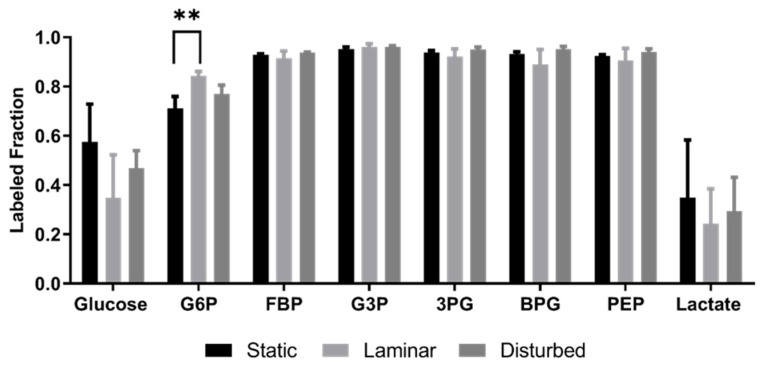
HUVECs adapted to steady laminar flow had similar labeled metabolite fractions to cells in static culture and oscillating disturbed flow, except for glucose-6-phosphate (G6P). Labeled glycolytic metabolite fraction (labeled/unlabeled) for HUVECs exposed to static culture, steady laminar flow, or oscillating disturbed flow for 24 h and then labeled with U-^13^C_6_-glucose for 5 min. Measured metabolites include glucose, glucose-6-phosphate (G6P), fructose 6-bisphosphate (FBP), glyceraldehyde-3-phosphate (G3P), 1,3-bisphosphoglycerate (BPG), phosphoenolpyruvate (PEP), and lactate. *n* = 3 samples per condition. ** *p* < 0.001.

We next analyzed the glycolytic metabolites in more depth, examining the intracellular total metabolite abundance (labeled + unlabeled; Figure 4), labeled metabolite abundance (Figure 5), and labeled metabolite fraction (labeled/unlabeled; Figure 6) for HUVECs exposed to 24 h of static culture, steady laminar flow, or oscillating disturbed flow. The intracellular total glucose pool was more than two times higher in HUVECs exposed to steady laminar flow (*p* < 0.05) compared to static culture, but for all other metabolites (except lactate), the total metabolite abundance was lower in cells exposed to steady laminar flow compared to cells exposed to either static culture or oscillating disturbed flow (Figure 4A). There was no statistically significant difference in total lactate abundance among cells in the different flow conditions.

We also examined the total metabolite abundances for the energy-providing nucleotides ATP, AMP, and ADP (Figure 4C). The monophosphate (AMP), diphosphate (ADP), and triphosphate (ATP) forms of adenosine were all reduced for HUVECs exposed to steady laminar flow as compared to static culture or oscillating disturbed flow, although only ATP reached statistical significance. We also calculated the adenosine ratios, since these can provide additional insight into the energy state of the cell. The ATP/ADP ratio was higher in the HUVECs exposed to steady laminar flow (~12) than in HUVECs exposed to static culture (~7) or oscillating disturbed flow (~5). The ATP/AMP ratio was similarly higher in the HUVECs exposed to steady laminar flow (~200) than in HUVECs exposed to static culture (~50) or oscillating disturbed flow (~25).

Labeled metabolite abundance, indicating metabolites derived from the ^13^C_6_ glucose added for 5 min after flow exposure, generally followed the same trends as for total metabolite abundance (Figure 5A). G6P, FBP, G3P, BPG, and PEP all showed lower metabolite abundance in HUVECs exposed to steady laminar flow. Only labeled glucose remained higher in cells exposed to steady laminar flow, but the difference was no longer statistically significant. Labeled BPG and PEP were also lower in cells exposed to oscillating disturbed flow as compared to static culture, which was similar to the trend observed for the total metabolite abundances. Labeled lactate decreased in cells exposed to steady laminar and oscillating disturbed flow as compared to static culture. HUVECs exposed to steady laminar flow had more than 60% fewer labeled glycolytic metabolites (Figure 5B) compared to cells in both static culture and oscillating disturbed flow (*p* < 0.001).

The labeled fraction, which is the labeled metabolite abundance divided by the total metabolite abundance, was not statistically different among groups for most metabolites because both total metabolite abundance and labeled metabolite abundance followed similar trends (Figure 6). The one exception was glucose-6-phosphate (G6P), which was higher in cells exposed to steady laminar flow than either static culture or oscillating disturbed flow (*p* < 0.001). This could indicate inhibition of the glycolytic enzyme downstream of G6P but upstream of FBP (e.g., PFK1). The labeled glucose fraction trended lower in HUVECs exposed to steady laminar flow, suggesting either reduced labeled glucose uptake or a larger intracellular unlabeled glucose pool. The labeled lactate fraction also trended lower in HUVECs exposed to steady laminar flow, similarly suggesting reduced labeled glucose metabolism into lactate or a larger intracellular unlabeled lactate pool. These data suggest that steady laminar flow decreased glucose flux through glycolysis, while also changing the intracellular G6P, glucose, and lactate pools.

**Figure 7:**
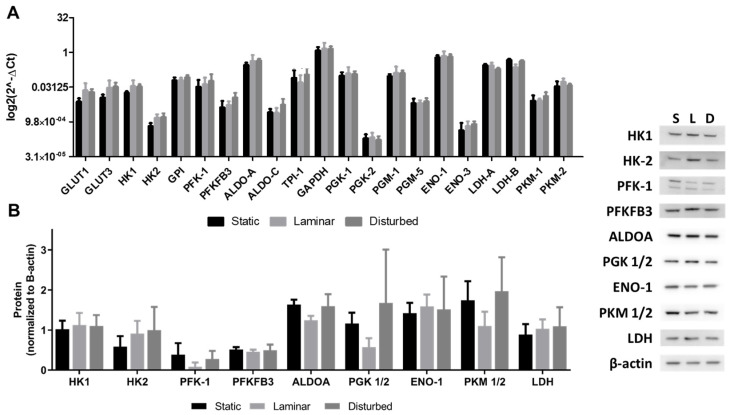
Glycolytic enzyme expression and protein levels did not change in HUVECs exposed to steady laminar versus oscillating disturbed flow. Relative (**A**) mRNA levels measured via RT-PCR, and (**B**) protein levels measured via Western blot of glycolytic enzymes in HUVEC exposed to static culture (S), steady laminar flow (L), or oscillating disturbed flow (D). GLUT1: glucose transporter 1, GLUT3: glucose transporter 3, HK1: hexokinase 1, HK2: hexokinase 2, GPI: glucose-6-phosphate isomerase, PFK-1: phosphofructokinase-1, PFKFB3: 6-phosphofructo-2-kinase/fructose-2,6-biphosphatase 3, ALDO-A: aldolase A, ALDO-C: aldolase C, TPI-1: Triosephosphate isomerase-1, GAPDH: Glyceraldehyde 3-phosphate dehydrogenase, PGK-1: phosphoglycerate kinase-1, PGK-2: phosphoglycerate kinase-2, PGM-1: phosphoglucomutase-1, PGM-5: phosphoglucomutase-5, ENO-1: enolase-1, ENO-3: enolase-3, LDH-A: lactate dehydrogenase A, LDH-B: lactate dehydrogenase B, PKM-1: pyruvate kinase-1, PFKM-2: pyruvate kinase-2. *n* = 3 samples per condition.

**Figure 8:**
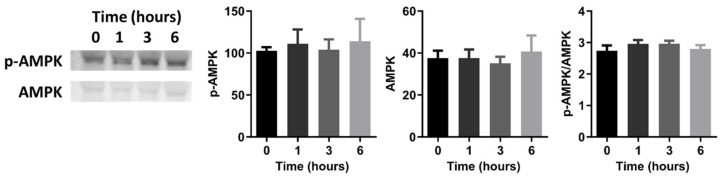
Phosphorylated AMPK did not change in HUVECs exposed to steady laminar flow. Western blot of phosphorylated AMPK (p-AMPK) and AMPK, and quantification of p-AMPK, AMPK, and p-AMPK/AMPK. *n* = 3 samples per condition.

To determine whether changes in the levels of glycolytic enzymes led to decreased glycolytic flux in endothelial cells in steady laminar flow, we measured glycolytic enzyme expression by qRT-PCR and protein levels by Western blot in endothelial cells exposed to different flow conditions. Glycolytic enzyme mRNA levels were largely similar for endothelial cells in static culture, steady laminar flow, or oscillating disturbed flow (Figure 7A). Similarly, there were few glycolytic enzyme protein changes in HUVECs exposed to steady laminar versus oscillating disturbed flow (Figure 7B). PFK-1 decreased in HUVECs exposed to steady laminar flow, although this was not statistically significant. AMP-activated protein kinase (AMPK), a highly conserved intracellular energy sensor, could also play a role in regulating endothelial cell metabolism in flow [24,25]. Indeed, AMPK has been shown to be phosphorylated within minutes to hours of flow initiation [26,27]. However, in our studies, we did not observe any significant increase in AMPK phosphorylation (Figure 8). We therefore concluded that the gene and protein expression of glycolytic enzymes by qRT-PCR and Western blot, respectively, were unlikely to play a large role in decreased glycolytic flux in HUVECs exposed to steady laminar versus oscillating disturbed flow.

**Figure 9:**
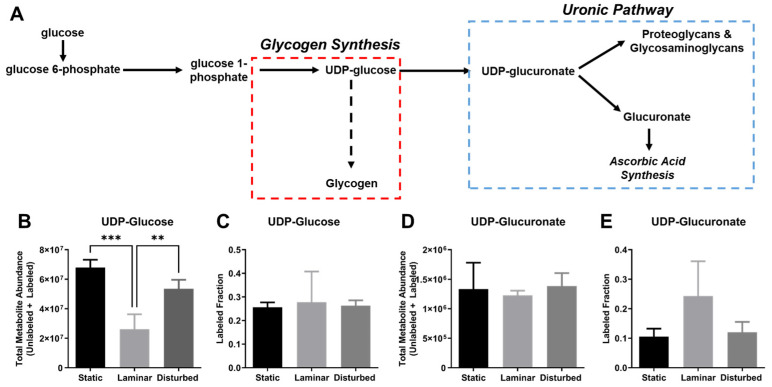
Glycogen synthesis and the uronic pathways may be differentially regulated in HUVECs exposed to steady laminar flow versus oscillating disturbed flow. (**A**) Diagram of glycogen synthesis and uronic acid pathways. (**B**) Total UDP-glucose metabolite abundance (labeled and unlabeled) and (**C**) labeled UDP-glucose fraction (labeled/unlabeled) in HUVECs exposed to 24 h static culture, steady laminar flow, or oscillating disturbed flow. (**D**) Total UDP-glucuronate metabolite abundance (labeled and unlabeled) and (**E**) labeled UDP-glucuronate fraction (labeled/unlabeled) in HUVECs exposed to 24 h static culture, steady laminar flow, or oscillating disturbed flow. *n* = 3 samples per condition. ** *p* < 0.001, *** *p* < 0.0001.

Finally, we examined the impact of the varied flow conditions on the glycolytic side branch pathways, glycogen synthesis, the uronic acid pathway, and the pentose phosphate pathway (PPP), which branch off glycolysis at glucose-6-phosphate (G6P; Figure 9A). UDP-glucose, which is a precursor for glycogen synthesis, showed lower total metabolite abundance in HUVECs exposed to steady laminar flow compared to HUVECs in either static culture and oscillating disturbed flow (*p* < 0.01, Figure 9B). However, the labeled fraction of UDP-glucose did not change (Figure 9C), suggesting that there was no change in ^13^C_6_-glucose incorporation into UDP-glucose for the different flow conditions. UDP-glucose can then become UDP-glucuronate and contribute to proteoglycan, glycosaminoglycan, and ascorbic acid synthesis in the uronic acid pathway. In this case, the total UDP-glucuronate abundance was similar for HUVEC in all flow conditions, while the labeled UDP-glucuronate fraction trended higher for HUVECs in steady laminar flow (Figure 9D,E). These data suggest changes in the glycogen synthesis and uronic acid pathways with flow.

**Figure 10:**
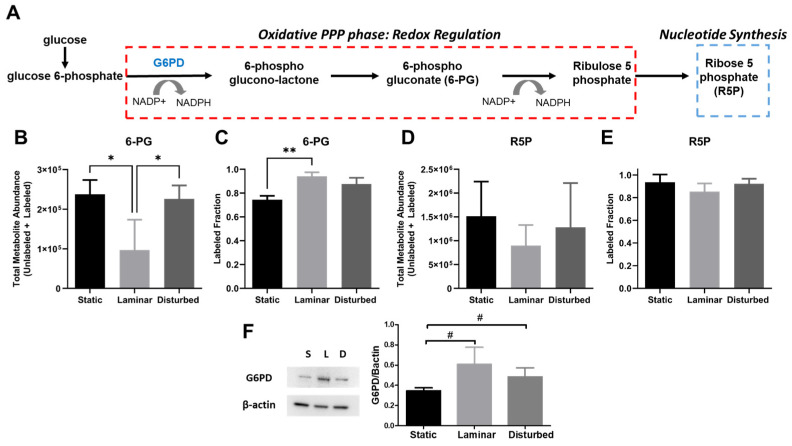
Glucose flux into the PPP increases in HUVECs exposed to steady laminar flow versus oscillating disturbed flow, which may reduce oxidative stress. (**A**) Diagram of the pentose phosphate pathway (PPP). (**B**) Total 6-phosphogluconate (6-PG) metabolite abundance (labeled and unlabeled) and (**C**) labeled 6PG fraction (labeled/unlabeled) in HUVECs exposed to 24 h static culture, steady laminar flow, or oscillating disturbed flow. (**D**) Total ribose-5-phosphate (R5P) metabolite abundance (labeled and unlabeled) and (**E**) labeled R5P fraction (labeled/unlabeled) in HUVECs exposed to 24 h static culture, steady laminar flow, or oscillating disturbed flow. (**F**) Western blot with quantification of glucose-6-phosphate dehydrogenase (G6PD), the rate-limiting PPP enzyme. *n* = 3 samples per condition. # *p* < 0.05, * *p* < 0.01, ** *p* < 0.001.

The PPP also branches off glycolysis at G6P and plays an important role in redox regulation and nucleotide synthesis (Figure 10A). Total 6-phosphogluconate (6-PG) decreased by more than 50% in HUVECs exposed to steady laminar flow, while the labeled 6-PG fraction increased by nearly 20% (Figure 10B,C). There were no statistically significant changes in ribose-5-phosphate (R5P; Figure 10D,E). These data suggest increased glucose flux into the PPP in cells exposed to steady laminar flow, specifically for redox regulation. Increased PPP flux is further supported by increased protein levels of glucose-6-phosphate dehydrogenase (G6PD), the rate-limiting PPP enzyme, in HUVECs exposed to steady laminar flow (*p* < 0.05), measured via Western blot (Figure 10F). These data suggest that endothelial cells in steady laminar flow increase PPP flux through increased G6PD protein, which could reduce oxidative stress.

## 3. Discussion

Glucose metabolism in endothelial cells exposed to oscillating disturbed flow has not been extensively studied, despite potential links between glycolysis and endothelial function [13]. We therefore elucidated how hemodynamics regulates endothelial glucose metabolism and found that endothelial cells exposed to steady laminar flow have decreased glycolytic flux compared to endothelial cells exposed to static culture or oscillating disturbed flow. Our data further show that alterations in glycolysis may also change glycolytic side branch pathways, such as the uronic acid pathway and the PPP. These studies highlight the need to further investigate endothelial glucose metabolic changes in oscillating disturbed flow, as they may be important in cell functions.

Our data show that endothelial cells exposed to oscillating disturbed flow had higher glycolytic activity than endothelial cells exposed to steady laminar flow. Elevated glycolysis is associated with endothelial proliferation and inflammatory response in vitro and in vivo, both of which are associated with atherosclerotic plaques [28,29]. For example, hypoxia-inducible factor 1-alpha (HIF-1α) was upregulated by 24 h of disturbed flow in human aortic endothelial cells (HAECs), and HIF1α, glycolysis enzymes, inflammatory genes and endothelial proliferation were all enhanced in low-shear-stress regions of porcine and murine arteries [28,29]. HAECs treated with siHIF-1α for 24 h prior to disturbed flow showed reduced changes in hypoxic and glycolytic genes [28]. Furthermore, PFKFB3-inhibited endothelial cells expressed lower VCAM-1, ICAM-1, and E-selectin upon inflammatory activation [19]. These studies support a link between glycolytic activity and inflammation. Future work could investigate how increased glycolytic flux in endothelial cells exposed to oscillating disturbed flow contributes to cell proliferation and inflammation.

There are several possible mechanisms by which glycolytic flux decreased in endothelial cells exposed to steady laminar flow for 24 h. First, endothelial cells in steady laminar flow took up less glucose, which would result in an overall decrease in downstream metabolites. Endothelial cells primarily take up glucose via the insulin-independent glucose transporter GLUT1. We did not measure any changes in GLUT1 levels; however, GLUT1 could be differentially translocated to the cell membrane in varied flow regimes [30]. Endothelial cells exposed to steady laminar flow may be more quiescent and have lower energy needs, which is supported by our mass spectrometry data showing lower ATP levels in endothelial cells exposed to steady laminar flow. Endothelial cells could also use other nutrients such as glutamine or fatty acids, which would also result in lower glycolytic activity.

Glycolytic enzymes could also play a role in decreased glycolytic flux in endothelial cells exposed to steady laminar flow. We observed a downward trend in PFK1 protein levels after flow exposure. Furthermore, the metabolite G6P had a higher labeled fraction in endothelial cells exposed to steady laminar flow. This could indicate an inhibition of glycolysis immediately downstream of G6P, which could occur due to lower PFK1 in endothelial cells in steady laminar flow. However, we did not observe a decrease in PFKFB3, which had previously been reported to decrease in endothelial cells adapted to steady laminar flow (20 dynes/cm^2^) for 72 h [22]. Twenty-four hours of flow may have been too short to measure altered gene expression or protein levels. Alternatively, enzyme activity, rather than expression or protein levels, may regulate glycolytic flux changes. For example, PFKFB3 kinase activity is upregulated by Ser461 phosphorylation in endothelial cells in cancerous tumors [25,31,32]. PFKFB3 is phosphorylated through several pathways, including AMPK, PKA, and PKC [33,34]. Shear stress rapidly activates these pathways, and pathway activation decreases below baseline after cells have adapted to steady laminar but not oscillating disturbed flow [35,36]. We did not observe any changes in phosphorylated glycolytic enzymes in our studies; however, this mechanism could be investigated further in the future.

Glycolytic enzyme activity can also be regulated by nucleotide products of glycolysis, for example, ATP, ADP, and AMP. PFK1 is allosterically inhibited by elevated ATP levels, which indicate an energy-rich state inside the cell. However, PFK1 is also allosterically activated by elevated AMP levels, which indicate depleted intracellular energy stores [37]. Our mass spectrometry data indicate that the total abundances of ATP, ADP, and AMP are all lower in endothelial cells exposed to steady laminar flow than either static culture or oscillating disturbed flow. The cellular energy state can also be determined through the ATP/ADP and ATP/AMP ratios, with a higher ratio indicating a higher energy state in the cell [24]. Both the ATP/ADP ratio and the ATP/AMP ratio were higher in the endothelial cells exposed to steady laminar flow than in endothelial cells exposed to static culture or oscillating disturbed flow. Thus, the high ratios of ATP and ADP to AMP could decrease glycolytic flux in endothelial cells in steady laminar flow by inhibiting PFK1 activity.

Finally, overall glycolytic flux could be altered by changes in flux down glycolysis side branch pathways, including glycogen synthesis and the PPP. We previously showed that flow decreases endothelial glucose flux down the hexosamine biosynthetic pathway, which impacts nitric oxide production [38]. In this study, we observed increased glucose flux down the uronic acid pathway, which could be used to create more proteoglycans and glycosaminoglycans. Indeed, shear stress can promote glycocalyx and extracellular matrix remodeling [39,40,41,42]. We also observed increased flux down the PPP, likely due to increased protein levels of the rate-limiting PPP enzyme G6PD. This could contribute to lower levels of oxidative stress in endothelial cells in steady laminar flow than cells in static culture or oscillating disturbed flow [43,44,45]. Thus, the metabolic shifts that occur with flow exposure may enable endothelial cells to remodel their microenvironment and maintain redox homeostasis.

In some cases, our YSI results differed slightly from the mass spectrometry data. We showed less glucose uptake by cells in steady laminar flow by YSI, yet higher total unlabeled glucose in cells exposed to steady laminar flow by mass spectrometry. There could be less glucose uptake by endothelial cells in steady laminar flow, but the glucose that is taken up is stored and not metabolized, therefore accumulating in the cell. YSI results also showed elevated lactate secretion in endothelial cells exposed to steady laminar flow compared to static culture. The mass spectrometry data showed a trend towards lower intracellular total, labeled, and unlabeled fractions of lactate in endothelial cells exposed to steady laminar flow. Our data potentially suggest that endothelial cells exposed to static culture and oscillating disturbed flow shuttle more pyruvate into the mitochondria for the TCA cycle, rather than sending pyruvate out of the cell as lactate [46]. Cells in steady laminar flow may instead shuttle more lactate out of the cell. Indeed, our preliminary data do suggest lower glucose metabolism in the TCA cycle in endothelial cells exposed to steady laminar flow, which could be examined in more detail in future studies.

While our study shows that glycolytic flux decreased in steady laminar but not oscillating disturbed flow in vitro, it is not without limitations. First, we only analyzed human umbilical vein endothelial cells. These effects should be confirmed in other endothelial cell types. All chronic flow studies were completed after 24 h of flow exposure since endothelial cells are morphologically adapted to flow at this time. Longer times could show additional changes in protein expression and quantity. We only quantified glycolysis by incubating cells for 5 min with ^13^C_6_-glucose. For future studies, longer incubation times could be investigated to examine oxidative phosphorylation. In addition, the protein levels of other important glycolytic regulators, such as HIF-1α, and the phosphorylation of key glycolytic proteins, should be measured. Finally, these in vitro results could be further validated with in vivo animal studies.

## 4. Materials and Methods

### 4.1. Endothelial Cell Culture

Human umbilical vein endothelial cells (HUVECs; Cell Applications, San Diego, CA, USA) were used for all experiments since they are a widely available, well-characterized cell type that is extensively used in endothelial metabolism research [12,13,19,20,22]. HUVECs (passages 5–10) were cultured in Endothelial Growth Medium-2 (EGM-2; Lonza, Basel, Switzerland) supplemented with 10% fetal bovine serum (FBS; Cytiva, Marlborough, MA, USA), 1% penicillin–streptomycin, and 1% L-glutamine (ThermoFisher Scientific, Waltham, MA, USA). All cells were maintained in a humidified environment at 37 °C and 5% CO_2_, with a medium change every 2 days. For flow studies, media were changed to Endothelial Basal Medium-2 (EBM-2; Lonza, Basel, Switzerland) supplemented with 10% FBS, 1% penicillin–streptomycin, and 1% L-glutamine 30 min prior to shear stress exposure.

### 4.2. Shear Stress Exposure

A custom-built cone-and-plate device was used to apply shear stress to HUVECs [38]. The device generates flow by rotating the cone around an axis perpendicular to the plate [47]. As long as the modified Reynolds number and cone angle are small, the shear stress can be determined from the following equation:

(1) $$\tau_{w}=\frac{\mu \omega }{\alpha }$$

where *μ* is the fluid dynamic viscosity, *ω* is the cone angular velocity, and *α* is the cone angle [48,49]. For steady laminar flow, the cone was programmed to rotate at a constant rate of 420 RPM, producing a shear stress of 20 dynes/cm^2^ at the endothelial cell surface [48]. For oscillating disturbed flow, the cone was programmed to rotate in an oscillatory manner to produce 4 ± 6 dynes/cm^2^ shear stress using the following equation:

(2) $$\omega \left(t\right)=7\frac{rad}{s}+(10.5\;\frac{rad}{s})\sin\left(2\pi ft\right)$$

where the amplitude shift 7 rad/s (85 RPM) equates to 4 dynes/cm^2^ and the sine wave amplitude 10.5 rad/s (126 RPM) equates to 6 dynes/cm^2^. The frequency was set at 1 Hz.

For each experiment, 17,000 cells/cm^2^ were seeded on a collagen Type 1-coated 60 mm culture dish (10 µg/mL at 37 °C for 3 h). Cells were cultured for 48 h and then assembled into the cone-and-plate device. In each experiment, three cone-and-plate devices were run in parallel. The entire cone-and-plate device was placed in a 37 °C, 5% CO_2_ incubator for the duration of each experiment. Static culture controls were placed in the same incubator.

### 4.3. Cell Viability and Proliferation Assays

Cell viability was assessed in HUVECs exposed to flow using a Live/Dead assay (L3224, ThermoFisher Scientific, Waltham, MA, USA) as per the manufacturer’s instructions. Briefly, 0.5 μM calcein-AM (live cells) and 1 μM ethidium bromide (dead cells) were added to samples in phosphate-buffered saline (PBS). After a 20 min incubation at room temperature, samples were imaged by confocal microscopy (LSM 700, Zeiss, Oberkochen, Germany). For proliferation, cells were fixed with 4% paraformaldehyde (P6148, Sigma-Aldrich, Burlington, MA, USA) for 20 min at room temperature, permeabilized with 0.1% Triton X-100 in PBS for 15 min at room temperature, and then blocked with 2% bovine serum albumin (BSA) in PBS for 30 min. Ki67 primary antibody (1:300; 701198; Thermofisher Scientific, Waltham, MA, USA) was then added to samples overnight at 4 °C. After washing, AlexaFluor 488 (1:300; A11001; ThermoFisher Scientific, Waltham, MA, USA) and Hoechst 33,342 solution (1:1000; 62249; ThermoFisher Scientific, Waltham, MA, USA) in 2% BSA in PBS were added to samples for 2 h at room temperature. Samples were then imaged by confocal microscopy. The images were obtained as z stacks of 5 μm steps, and all z planes were compressed into a single plane using the Extended Focus command in Volocity 6.3 cell imaging software (Perkin Elmer, Hopkinton, MA, USA). HUVEC viability and proliferation were quantified in ImageJ via the cell counter analyzer plugin.

### 4.4. YSI Bioanalysis

After HUVECs were exposed to 24 h steady laminar flow or oscillating disturbed flow as previously described, cells were given fresh EBM-2 for 3 h. A conditioned media sample was then collected and centrifuged at 1000 RPM for 5 min to remove debris. The supernatant was analyzed on the YSI 2950 analyzer (Xylem, Cheverly, MD, USA), following instrument calibration and precision verification using a standard with known glucose and lactate levels. Glucose consumption and lactate production were calculated based on glucose and lactate measured in experimental and control media samples. All samples were run in triplicate, and the average of the analytic replicates was used for the final analysis.

### 4.5. Mass Spectrometry

Endothelial metabolites were measured via ^13^C_6_-glucose liquid chromatography–mass spectrometry (LC-MS). HUVECs were exposed to different flow conditions for 24 h as described, after which 5 mM ^13^C_6_-glucose (CLM-1396; Cambridge Isotope Laboratories, Tewksbury, MA, USA) in glucose-, glutamine-, and pyruvate-free DMEM 1X (A14430-01; ThermoFisher Scientific, Waltham, MA, USA) supplemented with 10% FBS, 1% penicillin–streptomycin, and 1% L-glutamine media was added to HUVEC for 5 min. This time point was selected to focus on glycolytic metabolites. The ^13^C_6_-glucose medium was then removed and 80:20 methanol/water (−80 °C, extraction solvent) was added to cells for 15 min at −80 °C. Cells were scraped in the extraction solvent and cell lysates pipetted into Eppendorf tubes. Samples were centrifuged at 16,000× *g* for 10 min at 4 °C to pellet debris. The supernatant was transferred to a new tube, desiccated under nitrogen gas flow, and re-dissolved in LC-MS-grade water. Metabolites were analyzed via reverse-phase ion-pairing chromatography coupled to an Exactive Orbitrap mass spectrometer (ThermoFisher Scientific, Waltham, MA, USA) following an established protocol [50].

### 4.6. Western Blot

Protein levels were determined by Western blot. Samples were prepared by scraping cells off the surface in ice-cold lysis buffer (20 mM Tris, 150 mM NaCl, 1% Triton X-100, 2 mM EDTA, 2 mM PMSF, 0.1% SDS, 2 mM Na_3_VO_4_, 50 mM NaF, 10% glycerol, complete protease inhibitor (Roche, Indianapolis, IN, USA), pH 7.4). Samples were then centrifuged for 10 min at 10,000× *g*, 4 °C to remove insoluble material. Cell lysates were normalized for protein content by BCA Assay (ThermoFisher Scientific, Waltham, MA, USA), separated by SDS-PAGE on a 4–12% Bis-Tris gel (ThermoFisher Scientific, Waltham, MA, USA), and transferred to a nitrocellulose membrane (ThermoFisher Scientific, Waltham, MA, USA). After blocking in 5% BSA, membranes were incubated with primary antibodies for hexokinase 1 (46695), hexokinase 2 (374091), phosphofructokinase-1 (166722), aldolase A (390733), phosphoglycerate kinase (48342), phosphoglucomutase-1 (373796), lactate dehydrogenase (133123), glucose-6-phosphate dehydrogenase (373886), and enolase-1 (100812, all from Santa Cruz), as well as PFKFB3 (13123S) and GFAT1 (5322, from Cell Signaling, Danvers, MA, USA) at a 1:1000 dilution overnight at 4 °C followed by the appropriate secondary horseradish peroxidase-conjugated antibody (1:2000; Promega, Madison, WI, USA) for 1 h at room temperature. β-actin (SC47778-C4, Santa Cruz, Dallas, TX, USA) was used as the loading control. Protein bands were detected using an enhanced chemiluminescence kit (Western Lightning, PerkinElmer, Waltham, MA, USA) and visualized with a Fluorchem digital imager (Alpha Innotech, San Jose, CA, USA). Band intensity was quantified using AlphaEase FC software (version 6.0.0).

### 4.7. Quantitative Reverse Transcription Polymerase Chain Reaction (qRT-PCR)

Metabolic enzyme expression was measured via qRT-PCR. Total RNA was extracted from cell samples using an RNeasy kit (QIAGEN, Germantown, MD, USA) following the manufacturers’ instructions. RNA concentration was measured using a Nanodrop ND1000 (ThermoFisher Scientific, Waltham, MA, USA) and was considered pure if the 260/280 and 260/230 ratios were ∼2. Samples were then stored at −80 °C until analysis. For qRT-PCR, 1 μg RNA per sample was combined with DNAse I Reaction Buffer I and DNAse enzyme in nuclease-free water. cDNA synthesis was performed using the High Capacity cDNA Reverse Transcriptase kit according to the manufacturer’s instructions (ThermoFisher, Waltham, MA, USA). SYBR Green PCR Master Mix was used to perform qPCR to quantify gene expression using 20 ng cDNA per reaction. All primers were custom-designed and synthesized by ThermoFisher Scientific (see Table 1 below). ∆Ct was used to calculate gene expression as a fold change of experimental Ct value compared to the house-keeping gene (β-actin) expression [51].

### 4.8. Statistical Analysis

Statistical analysis was performed with GraphPad Prism (GraphPad, Boston, MA, USA). Each flow experiment was performed in duplicate or triplicate and repeated three times. Comparisons between two groups were analyzed by Student’s *t*-test, and comparisons among multiple groups were analyzed by one-way analysis of variance (ANOVA) with a Dunnett post hoc test. Statistical significance is indicated by # *p* < 0.05, * *p* < 0.01, or ** *p* < 0.001.

## 5. Conclusions

We now show that hemodynamics regulates glycolysis and glycolytic side branch pathways. Steady laminar flow decreased endothelial cell glucose consumption and glycolytic flux, while oscillating disturbed flow did not. Changes in glycolytic side branch pathways could support glycocalyx and extracellular matrix remodeling, as well as reduced oxidative stress, in endothelial cells in steady laminar flow. These data show that metabolism could impact phenotypic changes that occur in endothelial response to flow.

## Figures and Tables

**Table 1:** PCR primer sequences.

Gene	Sequence (Forward)	Sequence (Reverse)
GLUT1	AAG GTG ATC GAG GAG TTC TACA	ATG CCC CCA ACA GAA AAG ATG
GLUT3	GCTGGGCATCGTTGTTGGA	GCACTTTGTAGGATAGCAGGAAG
HK1	TCCGTAGTGGGAAAAAGAGAA	GACAATGTGATCAAACAGCTC
HK2	TAGGGCTTGAGAGCACCTGT	CCACACCCACTGTCACTTTG
GPI	GGAGACCATCACGAATGCAGA	TAGACAGGGCAACAAAGTGCT
PFK-1	GGTGCCCGTGTCTTCTTTGT	AAGCATCATCGAAACGCTCTC
PFKFB3	AAGCAGTACAGCTCCTACAACT	CTTCTTTCGCCAGGTAGCTTT
ALDOA	ATGCCCTACCAATATCCAGCA	GCTCCCAGTGGACTCATCTG
ALDOC	TGCTGATGACCGTGTGAAAAA	CGGACGAAGGGAACACCAT
TPI1	AGTGACTAATGGGGCTTTTACTG	GCCCAATCAGCTCATCTGACTC
GAPDH	ATGGGGAAGGTGAAGGTCG	GGGGTCATTGATGGCAACAATA
PGK1	GACCTAATGTCCAAAGCTGAGAA	CAGCAGGTATGCCAGAAGCC
PGK2	TTGACGAGAACGCTCAGGTTG	ACGGCCCATTCCAAACAATTAG
PGM1	GTGCAGAAGAGAGCGATCCG	CGGTTAGACCCCCATAGTGC
PGM5	TCGTCCATTCGTCTATGACGC	GGCTTCCAATGAGACACGG
ENO1	TGGTGTCTATCGAAGATCCCTT	CCTTGGCGATCCTCTTTGG
ENO3	TATCGCAATGGGAAGTACGATCT	AAGCTCTTATACAGCTCTCCGA
LDHA	TTGACCTACGTGGCTTGGAAG	GGTAACGGAATCGGGCTGAAT
LDHB	CCTCAGATCGTCAAGTACAGTCC	ATCACGCGGTGTTTGGGTAAT
PKM1	ATAGCTCGTGAGGCTGAGGCAGCCA	ACTCCGTCAGAACTATCAAAGCTGCT
PKM2	TGAGGCAGAGGCTGCCATCTACCAC	TGCCAGACTTGGTGAGGACGATTATG
GFAT1	GCAAGCAGTTGGCACAAGG	CTCCACTGCTTTTTC TTCCAC
G6PD	CCGTCACCAAGAACATTCACG	GGACAGCCGGTCAGAGCTCT
ACTB	GAGCGCGGCTACAGCTT	TCCTTAATGTCACGCACGATTT

## Data Availability

The data presented in this study are available on request from the corresponding author.
